# The Impact of Bilingualism on Working Memory: A Null Effect on the Whole May Not Be So on the Parts

**DOI:** 10.3389/fpsyg.2016.00265

**Published:** 2016-02-25

**Authors:** Noelia Calvo, Agustín Ibáñez, Adolfo M. García

**Affiliations:** ^1^Institute of Philosophy, School of Philosophy, Humanities and Arts, National University of San JuanSan Juan, Argentina; ^2^Faculty of Psychology, National University of CórdobaCórdoba, Argentina; ^3^Laboratory of Experimental Psychology and Neuroscience, Institute of Translational and Cognitive Neuroscience, INECO Foundation, Favaloro UniversityBuenos Aires, Argentina; ^4^National Scientific and Technical Research CouncilBuenos Aires, Argentina; ^5^Universidad Autónoma del CaribeBarranquilla, Colombia; ^6^Department of Psychology, Universidad Adolfo IbáñezSantiago, Chile; ^7^ARC Centre of Excellence in Cognition and its DisordersSydney, NSW, Australia; ^8^UDP-INECO Foundation Core on Neuroscience, Diego Portales UniversitySantiago, Chile; ^9^Faculty of Elementary and Special Education, National University of CuyoMendoza, Argentina

**Keywords:** bilingualism, bilingual advantage, executive functions, working memory, L2 proficiency, simultaneous interpreting

Abundant research has examined the relationship between bilingualism and working memory (WM), a system that keeps information accessible while dealing with concurrent processes, distractions, or attention shifts (Baddeley and Hitch, 1974; Engle et al., 1999; Conway et al., 2002). Some studies have reported no WM differences between bilinguals and monolinguals (Bialystok et al., 2008; Feng, 2009; Bialystok, 2010; Namazi and Thordardottir, 2010; Bonifacci et al., 2011; Engel de Abreu, 2011), leading top scholars to maintain that this domain is impervious to bilingualism. For instance, Bialystok (2009) first claimed that WM is indifferent to the development of a non-native language (L2). Later, she slightly reframed her position, stating that WM is only occasionally enhanced by the bilingual experience (e.g., Bialystok et al., 2009, 2012). Likewise, in another study, Engel de Abreu (2011: p. 6) concluded that “bilingual experience does not seem to convey any advantage in working memory abilities,” which aligns with recent criticism on the very notion of bilingual benefits (Duñabeitia and Carreiras, 2015; Calvo et al., 2016; Paap et al., 2016).

However, there is no shortage of evidence for enhanced WM in bilinguals. While full-blown WM advantages have been only sparsely reported, several studies yielding no *overall* benefits did find such effects in specific tasks or conditions. This is also true of comparisons between bilingual groups who daily exert different levels of demand on their WM systems (in particular, simultaneous interpreters vs. non-interpreting bilinguals). These findings indicate that WM is not *completely* unaffected by the distinctive executive demands of bilingualism. Instead, they suggest that a bilingual advantage may indeed exist in *some aspects* of WM, as we argue below.

The hypothesis underlying the field is that cognitive skills developed to cope with the demands of controlling two languages generalize to more efficient processing in executive domains, including WM. Relevant evidence is typically garnered as follows. First, two sociodemographically matched samples are recruited, one comprising bilinguals and the other composed of monolinguals—alternatively, these could be interpreters and non-interpreters. A set of tasks (including WM paradigms) are then administered to both groups, and their respective results are compared. Crucially, WM tasks vary widely across studies, as they involve different stimuli, procedures, and presentation modalities.

Within that literature, some studies reported concrete advantages for bilinguals. For instance, Bialystok et al. (2004) compared bilingual and monolingual adults (aged 30–80) in three different studies using a non-verbal Simon task. Overall, bilinguals outperformed monolinguals when WM demands were high, and the extent of the difference was proportional to age. Further evidence for a bilingual WM advantage was reported by Morales et al. (2013) in two experiments with children. To this end, the authors used a Simon-type task and a visual-spatial task. Their overall results showed that bilinguals surpassed monolinguals in all the conditions involving high WM and executive demands. Similarly, the bilingual children studied by Blom et al. (2014) showed better performance in visuospatial (Dot Matrix/Odd-One-Out) and verbal (Forward Digit Recall/Backward Digit Recall) WM tests when vocabulary was controlled for, especially in tasks that involved processing and not just storage.

Moreover, studies often cited as disconfirmatory evidence have actually reported enhanced performance by some bilingual groups under specific conditions. Feng (2009) presented various WM tasks to monolinguals and bilinguals from two age groups: children and adults. Despite null results in most conditions, a general bilingual advantage was observed in a spatial WM task (recalling the position of randomly ordered items). A similar result was reported by Bialystok et al. (2008), who evaluated bilingual and monolingual younger and older adults. In this case, participants completed different WM, lexical retrieval, and executive control tasks. While the adult groups showed no significant WM advantages, this effect did emerge for younger bilinguals in a Corsi Block task. Also, Namazi and Thordardottir (2010) compared the performance of young bilingual and monolingual children through assessments of verbal short-term memory, verbal WM, visual WM, and visual controlled attention. Although both language groups performed similarly in most tasks, bilinguals showed positive correlations between visual WM and attentional control skills. Finally, Bonifacci et al. (2011) tested bilingual and monolingual children with a choice reaction-time task, an anticipation task, a go/no-go task, and two WM tasks (numbers and symbols). In this case, only bilingual infants were faster in a visual anticipation task calling on WM resources. In sum, even those studies which failed to find overall WM advantages did report such an effect under certain circumstances.

In this sense, most studies have explored the issue using words or digits as stimuli (e.g., Bialystok, 2010; Engel de Abreu, 2011). Given that bilinguals generally have more difficulty than monolinguals in word processing (Bialystok et al., 2009), tasks with high verbal requirements may not be well suited to test the bilingual WM advantage hypothesis. Indeed, as seen above, WM tasks employing (non-verbal) visual stimuli have yielded consistent advantages for bilinguals.

Two views may account for this pattern. On the one hand, the bilingual experience may selectively enhance a visually-specialized subcomponent within WM. This possibility is compatible with Baddeley's model (Baddeley and Hitch, 1974; Baddeley, 2000), which posits that WM comprises a visuospatial sketchpad, separate from the so-called phonological loop. Moreover, it aligns with meta-analytic data indicating that the development of specific components of WM may be differentially associated with L2 proficiency (Linck et al., 2014). On the other hand, it may be that an undivided WM interacts with several systems in long-term memory. Those systems which are inherently weakened by bilingualism—in particular, verbal processing (Bialystok, 2009)—would carry over their processing disadvantages to any task which taps into them, including WM.

Note that executive skills needed to direct visual attention to location and space may be honed by increased language processing demands. In fact, attentional control mechanisms are essential to process visual (Chun and Wolfe, 2001) and verbal (Bialystok and Cummins, 1991) information. Moreover, the attentional control processes of WM may account for individual differences in the bilingual literature (Linck et al., 2014). In this respect, modality-specific bilingual advantages in WM may be related to increased attentional skills. Recent evidence supports this conjecture. Tse and Altarriba (2014) assessed bilingual children with varied proficiency levels through the Simon task (Simon/Simon switching) and an operation-span WM task. More proficient bilinguals showed better conflict resolution and WM capacity when the tasks demanded more attentional control.

Finally, if the proposed effects stem from increased control demands during bilingual processing, they should be greater in bilinguals who daily face particularly stringent processing conditions, such as simultaneous interpreters (García, 2014). Relationships between WM and interlingual processing skills have been reported in studies which did not consider interpreters. For example, Kroll et al. (2002) compared word naming and translation performance between native English speakers with different levels of L2 competence. In addition to the main finding of the study (better performance for the more fluent group), a positive correlation was found between the participants' WM and their translation performance. Such a result fits well with meta-analytic evidence that WM is robustly associated with L2 processing/proficiency outcomes (Linck et al., 2014). In light of these findings, it is also worth considering comparisons between professional interpreters (whose language processing is repeatedly subject to high WM demands) and non-interpreter bilinguals—an empirical corpus that previous discussions have mostly neglected.

Bajo et al. (2000) assessed lexico-semantic, comprehension, and WM abilities in professional interpreters, interpreting students, non-interpreter bilinguals, and monolinguals. The interpreters showed increased WM spans for digits and words, in addition to faster categorization, reading, and lexical access skills. Interpreters also showed increased abilities in other studies tapping WM storage through visual span tasks (Christoffels et al., 2006; Yudes et al., 2011). For instance, Christoffels et al. (2006) compared language and WM skills among professional interpreters, bilingual university students, and highly proficient L2 teachers. The interpreters outperformed both other groups in WM measures, including word span and reading span—for a fuller discussion, see García (2014).

Moreover, those advantages have been repeatedly observed in tasks involving verbal stimuli. Thus, while WM enhancements led by bilingualism proper (as opposed to monolingualism) may be more pervasive in (non-verbal) visual tasks, those guided by differential processing skills between bilingual groups could possibly manifest in other domains. Indeed, the meta-analysis by Linck et al. (2014) revealed that positive correlations between L2 proficiency and WM may be more pronounced for verbal than non-verbal measures of the latter domain.

In sum, specific aspects of WM may actually be enhanced by the bilingual experience. Discrepant results seem to reflect methodological differences among the studies, especially in terms of task- and stimulus-related variables. Specifically, failure to observe WM differences between bilinguals and monolinguals in most previous studies may be explained by the use of verbal stimuli, given that bilingualism seems detrimental to vocabulary skills. Future studies should evaluate which particular components within WM functioning are sensitive to the effects of bilingualism. For instance, it would be useful to assess whether bilingualism enhances the attentional components of WM in a stimulus- and modality-independent fashion.

To conclude, WM is a complex domain both in its internal configuration and in its connections to other cognitive systems. Bilingualism may not enhance WM function at large, but it may improve certain aspects of it. Whether such selective advantages correspond to improvements in mechanisms within WM remains to be empirically determined. However, extant evidence suffices to raise a word of caution: failure to observe an effect in certain aspects of a function should not be automatically taken as evidence for a null effect in all of its components. Further research on the distinctive aspects of bilingualism might benefit from this general premise.

## Author contributions

Overall idea: NC, AG. Literature review: NC, AI, AG. Manuscript elaboration: NC, AI, AG.

### Conflict of interest statement

The authors declare that the research was conducted in the absence of any commercial or financial relationships that could be construed as a potential conflict of interest.
